# Identification of spore-forming bacteria isolated from contaminated Lowenstein Jensen media and effectiveness of Vancomycin to reduce Mycobacterial culture contamination in Burkina-Faso

**DOI:** 10.1038/s41598-019-43662-0

**Published:** 2019-05-10

**Authors:** Antoinette Kaboré, Juliette Tranchot-Diallo, Hervé Hien, Ousséni Zouré, Dezémon Zingué, Adama Sanou, Michel Kireopori Gomgnimbou, Géraldine Daneau, Georges Anicet Ouédraogo, Nicolas Méda, Lassana Sangaré

**Affiliations:** 1 Département des Sciences Biomédicales, Centre MURAZ, Bobo- Dioulasso, Burkina Faso; 2 UFR/ST, Université Nazi BONI, Bobo-Dioulasso, Burkina Faso; 3 Département de Santé Publique, Centre MURAZ, Bobo-Dioulasso, Burkina Faso; 4 Sciensano, Bruxelles, Belgium; 5 Département de Bactériologie-Virologie, CHU Yalgado OUEDRAOGO, Ouagadougou, Burkina Faso; 6 UFR/SDS, Université Ouaga I Pr Joseph Ki ZERBO, Ouagadougou, Burkina Faso

**Keywords:** Microbiology techniques, Clinical microbiology

## Abstract

The type of commensal microorganisms can influence the efficiency of sputum decontamination for TB diagnosis. A basic characterization of contaminants from LJ contaminated media showed that Gram positive Spore Forming Bacteria (SFB) were the major contaminants. This study aims to identify the species of this contaminants and to evaluate the effectiveness of VCNT at 10 µg of vancomycin to reduce mycobacterial culture contamination mainly linked to SFB. Fifty-three SFB isolated between February 2016 and May 2017 were used. The effectiveness of LJ with VCNT at 10 µg of Vancomycin were evaluated with sputum collected in the same period. SFB had been stored at −20 °C and identified after subculture onto 5% sheep blood Columbia agar and incubated at 37 °C during 24 h. Bacteria cells and isolated colonies were described. API 50CH/B was performed and MALDI-TOF MS was used for external quality control. Thirty- five (66%) isolates representing 4 genera (*Bacillus, Paenibacillus, Brevisbacillus and Lysinibacillus*) including 10 species were identified. The most important species were *Bacillus cereus* (30%) and *Bacillus licheniformis* (21%). Eighteen (34%) isolates were non-reactive *Bacillus*. The overall contamination rate on LJ with VCNT at 10 µg of vancomycin was statistically lower than which without VCNT (18.7% versus 43.8%) (p = 0.01). The most important SFB identified were B*. cereus and B. licheniformis*. Almost all identified strains were similar to those currently isolated in fermented traditional food suggesting in part food related contaminants. VCNT containing 10 µg of vancomycin is a good alternative method to reduce mycobacterial culture contamination.

## Introduction

Culture on solid media remains the gold standard for detecting TB and drug sensitivity testing^1,2^. However, the effectiveness of culture systems is greatly undermined by contamination with other bacteria and fungi^3–5^. The sputum culture process on LJ media, include a step of decontamination in order to eliminate contaminating microorganisms while protecting mycobacteria^6^. But the type of the contaminant microorganisms can influence the efficiency of the decontamination^7^. Indeed, the presence of an external envelope for Gram negative bacteria, of a wax coat for mycobacteria or a sporale coat for spore forming bacteria (SFB) lead to a variability of sensibility to biocides within the microorganisms^8^.

The literature reports little and different data about contaminants from LJ media. Studies carried out in Ireland, in 2011, and in Uganda in 2014, showed that a Gram negative bacteria (Pseudomonas *aeruginosa* (47.2%), *Serratia marcescens* (13%)), and Gram positive bacteria excluding SFB (*Staphylococcus spp* (50%) and *Streptococcus spp* (16%)), were respectively the main contaminant species isolated^9,10^. A few studies involved mycobacterial culture from different specimen (sputum, liquid gastric) on different media (liquid and solid) reported the presence of SFB among contaminants in mycobacterial cultures^9,11–14^. But possibly due to the difference in the context, none of them mentioned SFB as major contaminants. Recently in 2017, a basic characterization of sputum culture contaminants in Burkina-Faso, showed for the first time that SFB from the genus of *Bacillus and related* were the major residual contaminants^15^. But no specific identification of SFB species has been carried out on these studies, excepted that of McClean *et al*.^9^. However, knowing the species to which these contaminants belong would provide information on their likely origin (maybe for prevention), and would help to understand and to resolve the contamination issue.

These contaminants, particularly resistant to biocides due to their ability to sporulate give for the first time, a stronger justification for high contamination rate of mycobacterial culture in Burkina Faso and also maybe in most resource limited sittings. Fortunately, vancomycin, generally inactive on mycobacteria is active against SFB^16,17^, therefore, it was a potential candidate to include in the antibiotic combination used as a selective supplement for culture media. Limited data have indicated that culture with mycobacterial Growth Indicator Tube (MGIT) supplemented with the Vancomycin, Amphotericin B and Nalidixic acid (VAN) combination and culture with Selective Kirchner medium supplemented with Vancomycin 10 mg/l (SKV) showed important reduction of culture contamination^18,19^. However, these studies were limited to liquid medium, less used in resources limited sitting because of the high costs associated with them. Therefore, we believe that sputum culture for mycobacteria, using the popular LJ medium, supplemented with Vancomycin at 10 µg, Colistin, Nystatin and Trimethoprim (VCNT) could contribute to reduce the culture contamination. The use of VCNT is doubly advantageous because of its availability in most African laboratories, and the specificity of its antibiotic combination that targets both SFB and other Gram negative, Gram Positive bacteria and fungi (knowing the poly-microbial character of sputum).

The objective of this study was to identify the species of SFB,to evaluate the effectiveness of VCNT to reduce mycobacterial culture contamination rate and to ensure the tolerance of mycobacteria and the susceptibility of SFB to VCNT.

## Results

### Phenotypic characterization of Spore forming Bactria

#### Morphology

**Figure 1:**
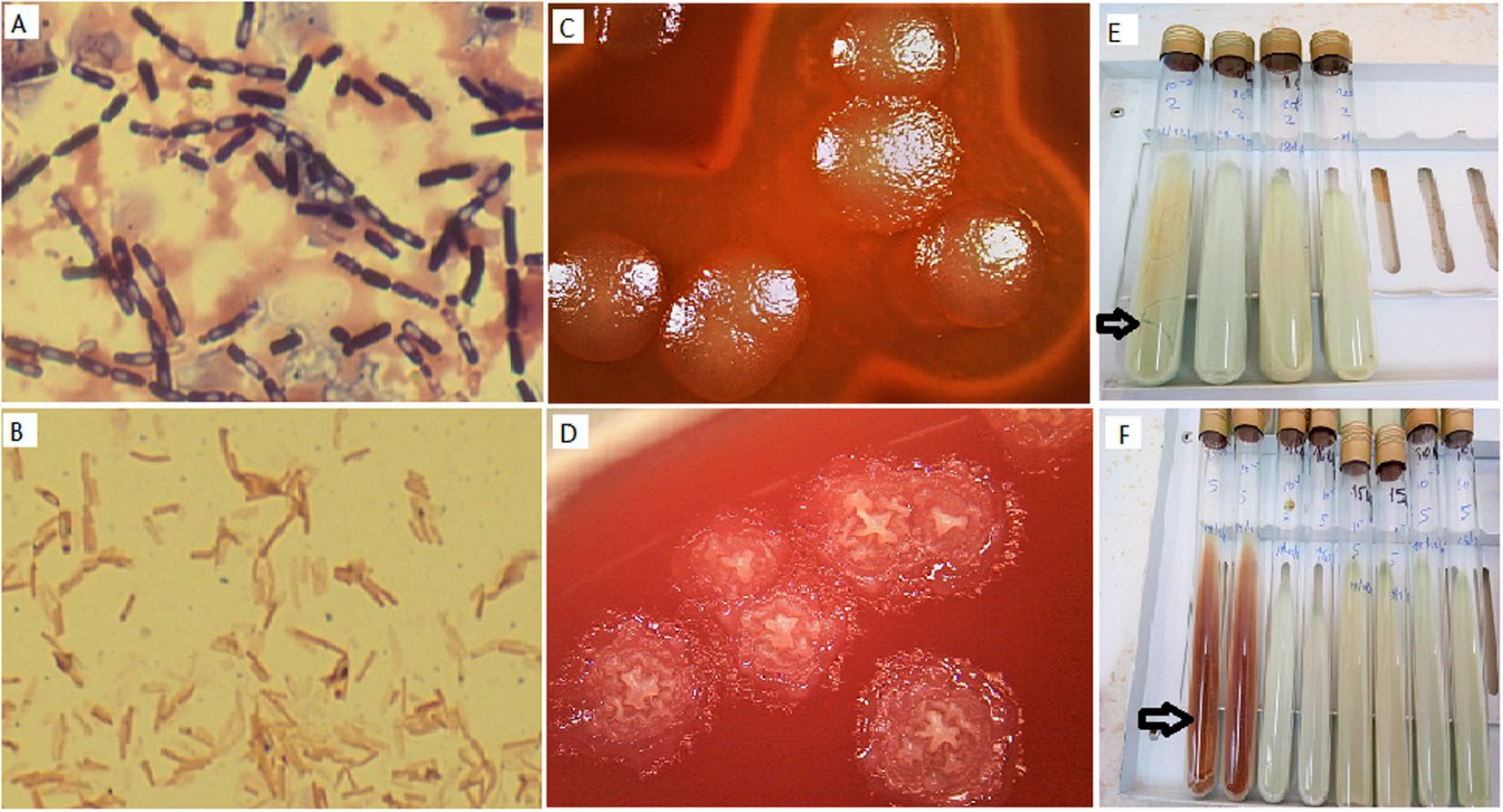
*B. cereus* and *B. licheniformis* cells, colonies and contamination aspect. *B. cereus*: Gram-positive rods, alone, in pairs and in chain with unwarping endospores representing bacteria cells of the group1 (**A**); Large, smooth, grey, regular, and Hemolytic colonies after 24 h of growth on 5% sheep blood agar (**C**); contamination aspect on LJ media (**E**). ***B. licheniformis***: Gram-positive rods, alone and in pairs (**B**); middle, rough. Creamy/grey, irregular and non- hemolytic colonies after 24 h of growth on 5% sheep blood agar (**D**); contamination aspect on LJ media (**F**).

Globally, microscopic examination of SFB colonies and cells showed 8 different morpho-groups. Their represented different size and forms of colonies, hemolytic and non-hemolytic, different size of rod-shaped bacteria, producing endospores of different forms, at different positions in bacteria cells (Table 1). All the isolates were catalase positive. The most important of which were the morpho-groups 1 and 2 with respectively 16 (30.18%) and 11 (20.75%) isolates (Table 1). The group 1 bacteria were Gram positive, large motile rods, are alone, grouped in pairs and in chain with ellipsoidal and cylindrical, central and subterminal spores which do not deforming the bacteria cells (Fig. 1A). They grow large, smooth, grey, regular and hemolytic colonies (Fig. 1C). The group 2 bacteria were Gram positive, middle or little motile rods, alone, in pairs, and in chain sometimes with ellipsoidal and cylindrical, central and subterminal spore which do not deforming the bacteria cells (Fig. 1B). They grow middle, rough creamy, irregular and non- haemolytic colonies (Fig. 1D).

**Table 1:** Phenotypic characteristics of spore forming bacteria strains isolated from contaminated LJ media.

Bacteria group	Colony description	Cell morphology	Gram	Spore	Cat*	oxy **	No. (%)
Group 1	Large, smooth, grey, regular, Haemolytic	Motile rods alone, in pairs and in chain	Gram positive	+ Ellipsoidal and cylindrical, Central and subterminal, not warping	+	+	16 (30.18)
Group 2	Middle, rough. creamy, irregular, non- hemolytic	Motile rods alone and in pairs and in chain	Gram positive	+ Ellipsoidal and cylindrical, Central and subterminal, not warping	+	+/−	11 (20.75)
Group 3	Middle, smooth, creamy, regular,	Motile rods alone and in pairs	Gram positive	+ Ellipsoidal, subterminal, warping	+	+	3 (5.66)
Group4	Large, mucosal, irregular, non- hemolytic	Motile rods alone and in pairs	Gram positive	+Ellipsoidal, subterminal, not warping	+	+	2 (3.77)
Group5	Large, smooth, grey, regular, Non-Haemolytic	Motile rods alone, in pairs and in chain	Gram positive	+ Ellipsoidal and cylindrical, Central and subterminal, not warping	+	+	1 (1.8)
Group6	Small, smooth, creamywhite, regular, non- hemolytic	Motile rods alone and in pairs and in chain	Gram positive	+ Ellipsoidal, Central and subterminal, not warping	+	+	1 (1.8)
Group 7	Large, mucous, transparent, irregular, non- hemolytic	Motile rods alone and in pairs	Gram positive	+ Ellipsoidal, sub terminal, not warping	+	+	1 (1.8)
Group 8	Middle/ Small, smooth, creamy, regular, non- hemolytic	Motile rods alone and in pairs	Gram variable	+ Ellipsoidal or round, Subterminal and terminal, warping	+	+	18(33.9 318)
Total							53 (100)

Cat*: Catalase test.

Oxy**: oxidase test.

#### Identification

Biochemical identification performed with the API 50 CHB system identified 35 of the 53 strains representing 3 genera (*Bacillus, Paenibacillus and Brevibacillus)* including 8 species. Thirty-four (64%) isolates were identified with more than 60% of similarity, one (2%) isolate with 58% of similarity, and 18 (33.96%) isolates which were unable to ferment any of the 49 carbohydrates present in the API 50CH/B strip were identified as non-reactive (Table 2). External quality control with the MALDI-TOF showed 87% (13/15) of accordance with the API 50CH/B. Only one discordance on specie level (*B. firmus* 58% similarity, identified as *B. cereus* by MALDI-TOF) was observed and one supplementary species was identify as *Lysinibacillus fusiformis* for one non-reactive isolate (Table 2). Finally, this study indicated the presence of 10 species (*B. cereus, B. licheniformis, Paenibacillus thiaminolyticus, Paenibacillus alvei, B. Subtilis/amyloliquefaciens, Brevibacillus laterosporus, B. firmus, B. anthracis, B. vallismortis and Lysinibacillus fusiformis)* (Table 2). The most important were *B. cereus* (30%) and *B. licheniformis* (21%). Around 69% of isolates from confirmed TB cases was formally identified against 55% for those from negative TB cases.

**Table 2:** Identification of species of spore forming bacteria isolates.

API 50 CH/B	Isolates Nb.	Carbohydrate fermented Nb.	Similarity % (Nb.)	MALDI-Tof		score
Bacteria species				Bacteria species	Isolates Nb.	
*B. Cereus1*	1	12	94.10(1)	*B. Cereus*	4	[2.00; 2.39]
*B.Cereus1*	11	13	71.40 (2), 72.80 (9)			
*B. Cereus2*	1	9	92.40(1)	*B. cereus*	3	
*B. Cereus2*	1	10	79.80(1)			
*B. Cereus2*	2	11	79 .3(1), 88 (1)			
*B. Licheniformis*	5	25	99.90 (3), 66.10 (2)	*Licheniformis*	4	[2.3; 2.47]
*B. Licheniformis*	2	26	99.80 (2)			
*B. Licheniformis*	2	27	99.90 (1),			
*B. Licheniformis*	2	28	99.90 (3) 100 (1)			
*Paenibacillus thiaminolyticus*	1	21	99.60 (1)	*Paenibacillus thiam*. Paenibacillus thiam./B. vallismortis* Paenibacillus thiam./B. cereus*	1	[2.10; 2.17]
*Paenibacillus thiaminolyticus*	1	23	99.90 (1)		1	
*Paenibacillus alvei*	1	26	99.90 (1)		1	
*B. Subtilis/amylo*	2	24	98.8 (1), 99.5 (1)	Not identified	1	NA
*B. anthracis*	1	10	83.10 (1)	Not identified	1	NA
B. firmus**	1	6	57.70 (1)	B. Cereus**	1	2
*Brevibacillus laterosporus*	1	10	76.6	Not identified	1	NA
Not reactive	18	0	NA	***Lysinibacillus fusiformis***	1	2.15

*Two mixed species were found in **MALDI-Tof** identification on the two isolates marked in bold and containing *Paenibacillus thiaminolyticus*.

**Different identifications were obtained from API and MALDI-TOF for the isolate marked in bold.

### LJ with VCNT effectiveness on clinical sputum culture contamination

The overall contamination rate on LJ with VCNT at 10 µg of vancomycin was statistically lower than which with LJ without VCNT (18.7% versus 43.8%) (p = 0.01). Negative culture was two time as great on LJ with VCNT as on LJ without VCNT (12. 5% versus 6. 2%) (Table 3).

**Table 3:** Comparison of sputum culture result on LJ with or without VCNT.

Sputum culture		LJ without VCNT			Total %	Chi2	p
		Nb of Not contaminated		Nb of contaminated			
LJ with VCNT	Nb of Not contaminated	Pos	Nég			4.9	0.011
	Pos	13	1	8	68.8		
	Nég	2	1	1	12.5		
	Nb of Contaminated	1	0	5	18.8		
	Total %	50	6.2	43.8	100		

Legend:

Nb: Number.

### Mycobacterium tolerance and spore forming Bacteria susceptibility to VCNT

#### Mycobacterium tolerance to VCNT

The CFUs number was uncountable (confluents and semi-confluents colonies) at 10^−1^ dilution for three (3/7) isolates of mycobacteria, as well as the BCG. For the remaining 4 isolates of which the CFUs was countable, the mean CFUs on LJ without VCNT and LJ with VCNT at 10, 15 and 30 µg of vancomycin were not different (mean CFUs = 95.5, 64, 44.5 and 33.5 respectively with p = 0.34, 0.34 and 0.32 respectively).

CFUs were countable for all isolates of mycobacteria at 10^−3^ dilution. The mean CFUs on LJ with VCNT at 10 µg of vancomycin compared to control were not significantly different (45.43 vs 31.14, p = 0.56; IC 95: −37.15-65.73), but it differ for LJ with VCNT at 15 and 30 µg of vancomycin (45.43 vs 16.85 and vs 3.71 respectively; p = 0.03 and p = 0.00 respectively).

#### Spore forming bacteria susceptibility to VCNT

After three days of incubation, 12/15 (80%) Bacillus have contaminated LJ without VCNT. We noted that any (0/15) Bacillus has contaminated LJ with VCNT at any concentration and inoculum dilution. After 7 days of incubation, all LJ without VCNT were contaminated. One B. cereus and one non-reactive Bacillus have contaminated LJ with VCNT at 10 µg of vancomycin for inoculum diluted at 10^−1^ and 10^−3^. This represented a low contamination rate of 13% that compared to our standard contamination rate was statistically different (13% vs 40%; p = 0.035; IC95: 3.73-37.88). One non-reactive Bacillus have also contaminated LJ with VCNT at 15 µg of vancomycin for both dilution (contamination rate: 7%). Compared to our standard contamination rate, the difference was statistically significant (p = 0.00; IC 95: 1.18-29.8). After 14 days of incubation, 3/15 Bacillus (two *B. cereus* and one non-reactive B.) have contaminated LJ with VCNT at 10 µg of vancomycin for both dilution (contamination rate of 20%). Compared to our standard contamination rate, the difference was not statistically significant (p = 0.11, IC: 7.04–45.18). No Bacillus have contaminated LJ with VCNT at 30 µg of vancomycin and the control Escherichia coli ATCC25922 (sensible to VCNT) have not grown on any LJ with VCNT. The general appearance of contaminants on media was characteristic of Bacillus groups. The *B. cereus* group was characterized by a stratal growth of brown-yellow color with a large liquefaction of media (cracked medium), those of *B. licheniformis* were also in a stratal growth, with a raspberry red pigment and a less significant liquefaction of the media (respectively Fig. 1E and Fig. 1F), the *Paenibacillus* were characterized by the turquoise blue turn of the media without liquefaction.

## Discussion

The objective of this study was to identify the species of SFB isolated as a major contaminants from LJ media during pulmonary tuberculosis (TB) diagnosis, to evaluate the effectiveness of VCNT to reduce mycobacterial culture contamination rate and to ensure the tolerance of mycobacteria and the susceptibility of SFB to VCNT.

The phenotypical description show that our major isolates are group 1 and group2. They are corresponding respectively to bacteria cells of group1 (sub- group A) and group 1 (sub- group B) described by Drobniewski *et al*., in 1993 representing *B. cereus* group and *B. subtilis group*^20^. According to Drobniewski, these group of *bacillus* are the most frequently isolated in clinic, so their isolation in sputum is not an exceptional occurrence.

The API 50CH/B identification indicate the presence of 8 species which the most important were *B. cereus* (30%) and *B. licheniformis* (21%) representing respectively morpho-group1 and 2 of Table 1. One important fact is that the positive reactions at 24 h for all *B. licheniformis* strains, are transformed to false-negative at 48 hours of incubation. Since many studies described the natural production of alkaline proteases by *B. licheniformis* strains^21^, this phenomenon is probably due to the neutralization of the acid by alkaline proteases produced. The API 50CH/B results were not in complete agreement with external quality control. Indeed, one SFB identified with low similarity rate (58%) as *B. firmus by* API50 CH/B was identified as *B. cereus* by MALDI-TOF-MS. According to current taxonomy, this specie is belong to *B. subtilis* group and discrimination with *B. cereus* should be made without trouble, but it is not the case here. Morphologically, Bacteria cell of this isolate looked like *B. cereus* but it fermented only 6 carbohydrates instead of 13 as the majority of *B. cereus*. Molecular identification would be necessary for clear identification. API 50CH/B failed to identify 18 (33%) heterogeneous isolates. Any of these strains has not fermented the 49 carbohydrates of the API 50CH / B and consequently, no similarity in species of *Bacillus and related* was able to be established. According to their morphology group 8 (Table 1), these bacteria belong to group II and group III of the classification of Drobniewski^20^. Unfortunately, almost all of these isolates was not analyzed with MALDI-TOF-MS method, what would maybe allowed to refine the identification of these non- reactive group of *Bacillus* and related. In spite of these limits, we were able to highlight the major spore forming bacteria.

*B. cereus* (30%) and *B. licheniformis* (21%) were the most important isolates identified in this study. These results were different to those of Ireland, and Uganda which respectively showed that Gram negatives bacteria (*Pseudomonas aeruginosa* 47.2% and *Serratia marcescens* 13%) and Gram positives bacteria (Staphylococcus *spp* 50% and *Streptococcus spp* 16%) were the major isolated contaminants^9,10^. Only 2% of *B. Licheniformis* was identified in Ireland study^9^. This difference could be explain by climatic, geographical and ethnic conditions (nutrition, genetic, immunities which lead to variability of the oral microbiome)^22^, mainly for Ireland study where the climate was tempered oceanic and ethnic conditions were more different to ours compared to Uganda closed to us. Indeed, the main nutritional condition is a strong argument for our results because we noticed that *Bacillus spp* are usually reported as starter in fermented foods, and are largely used in African and particularly in Burkina Faso diet^23–27^. So their isolation in sputum culture in our country, should not be exceptional.

We noted a wide similarity (74%) between the species that we identified and those identified as a starter in fermented foods in Burkina-Faso. It was substantively *B. subtilis B. licheniformis*, *B. cereus*, *B. firmus* and *B. borstelensis*^25,26^. This indicates that food may plays at least in part, an important role in sputum culture contamination by transferring these microorganisms in oropharynx or in pulmonary ecosystems. Even if we don’t know the interactions which can facilitate this colonization, we know that their isolation in contaminated LJ media is linked to contributing factors. Indeed, the use of sodium hydroxide during decontamination process of sputum could be the way of chemo-resistant microorganism’s selection. In fermented foods the long boiling time of *Parkia biglobosa* and *Hibiscus sabdariffa* seeds during Bikalga and soumbala (fermented foods) preparation are the ways of these thermos-tolerant microorganisms selection.

Our results raises one important question: the real role of the presence in sputum of *B. cereus*, *B. licheniformis* and *Subtilis* the most frequently bacillus implicated in human infection^28^. Many arguments suggest that the precautionary principle must be applied and that clinicians and clinical microbiologists must take care, instead of considering these bacteria as being simply contaminants. First, already in 1993, Drobniewski *et al*. asserted that most of the important strains of *Bacillus* isolated clinically belonged to *Cereus* and *Subtilis* group^20^ and therefore in the specific case, this presence maybe is not fortuitous. Indeed, *Bacillus cereus* is well known as a cause of food poisoning, and *B. licheniformis* has occasionally been isolated from cases of food associated illness^29,30^. In addition to food poisoning, *B. cereus* and *B. licheniformis* in recent years have been increasingly implicated in a wide range of infections including abscesses, bacteremia/septicemia, wound and burn infections, ear infections, endocarditis, meningitis, ophthalmitis, osteomyelitis, peritonitis, and respiratory and urinary tract infection*s* in both immunologically compromised and immunocompetent individuals^28,30–34^. Moreover, *paenibacillus* and *brevisbacillus* identified also in our study were sporadically isolated in clinical sample (Blood, urine, drainage, bronchial aspirate.^17,35,36^. Lastly, *B. anthracis*, one of the most pathogenic *Bacillus*, involved in human infections, was also identified on one isolate belonging to TB negative case. Secondly, although it is not the objective of this current study, characters of virulence as hemolysis and protease secretion were highlighted on most of our isolate. Finally, current evidence suggests that the lung microbiome is a potential modifiable risk factor for TB infection and disease^37^. All this arguments suggest a possible association between these SFB (particularly *B. cereus, B. licheniformis, Paenibacillus B. anthracis)* and the pulmonary TB or pneumonia in general. Future studies are necessary to confirm this hypothesis.

Our second objective was to check how to overcome of these chemo-resistant contaminants in the sputum culture process by using VCNT at 10 µg of vancomycin and to ensure proper mycobacterial tolerance and susceptibility of SFB to VCNT. The sputum culture on LJ with VCNT at 10 µg of vancomycin showed a significantly lower contamination rate and a better recovery rate of mycobacteria compared to that on LJ without VCNT. Although widely used in classic bacteriology laboratories, VCNT to our knowledge has never been used as a selective supplement in LJ media. However, another selective supplement (VAN: Vancomycin, nalidixic acid and nystatin) containing vancomycin at 10 µg has been used with liquid media for gastric aspiration culture for mycobacteria, and has similarly shown a significant reduction of culture contamination^18^. Despite the small number of samples tested, this check suggests that the selective LJ medium with VCNT could be used for the isolation of mycobacteria from sputum, mainly for laboratories that are facing high levels contamination linked to SFB. However, the contamination rate remains above the acceptable rates (5–10%). Therefore, it was important to study the tolerance of mycobacteria and the susceptibility of SFB to VCNT from the perspective to increase the concentration of VCNT.

Compared to control, the mycobacteria showed good tolerance at all concentrations of VCNT for inoculum diluted at 10^−1^ (p ≥ 0.32), But for those diluted at 10^−3^, this tolerance is maintained only for the VCNT at 10 µg of vancomycin but not for the VCNT at 15 or 30 µg of vancomycin (respectively: p = 0.56; p = 0.03 and p = 0.00). This other check suggests that using the LJ with VCNT at 10 µg of vancomycin would not affect the isolation of mycobacteria, as opposed to the LJ with VCNT at 15 or 30 µg of vancomycin, where there might be a real risk for mycobacterial recovery especially for paucibacillary sputum. However, the use of VCNT at 15 µg vancomycin could be considered for strongly positive smears.

For SFB susceptibility, our result showed increasing susceptibility of SFB depending on the VCNT concentration. Thus, contamination rates on LJ with VCNT at 10 µg of vancomycin, were 0%, 13% and 20% after 3, 7 and 14 days of incubation respectively, 0%, 6% and 6% for LJ with VCNT at 15 µg of vancomycin and 0% for LJ with VCNT at 30 µg of vancomycin for any incubation time. Even if LJ with VCNT at 15 µg or 30 µg of vancomycin allow better reduction of contamination, the results of tolerance and susceptibility taken together, suggest that the concentration of VCNT at 10 µg of vancomycin is that which allows both optimal preservation of mycobacteria and optimal removal of SFB. This concentration of VCNT allow an important reduction of contamination linked to SFB (20%) after 14 days of incubation compared to our standard contamination rate (around 40%). Nevertheless, the observed level of resistance to VCNT induces contamination rates above acceptable levels recommended by the WHO (5–10%). In fact, this method could just be used as an alternative one until the development of a more efficient methods. One of the potential candidates could be a method that initiate germination, blocks sporulation and would therefore target the vegetative form which is the Achilles heel of SFB.

When the incubation time of SFB susceptibility testing did not exceed three days, all the isolates were susceptible to VCNT at 10 µg of vancomycin. This results was similar to studies on clinical Bacillus and on African traditional food starters^38–40^. But increased incubation time appears to reduce effectiveness of VCNT at 10 µg of vancomycin. This is a concern, but contamination is expected to stabilize after 14 days of incubation, because this deadline will allow sufficient time for the majority of mycobacteria to begin to grow. We noted that the contamination appearances were specific, particularly for *B. cereus*, *B. licheniformis* and *Paenibacillus*. This specificity of the appearance of contamination could serve as a tool for predicting the types of contaminants.

In summary, this check indicated that VCNT at 10 µg of vancomycin is a good alternative to reduce contamination of sputum culture, particularly those linked to SFB. Increasing the VCNT concentration for reducing contamination is not an option for paucibacillary sputum but could be used for strongly positive smears. A multicenter evaluation is necessary to sharpen and confirm all these results mainly for resource-limited settings where contamination issue are frequent.

## Conclusion

The identification of isolated SFB from contaminated LJ media belonging to the *Bacillus, Paenibacillus, Brevibacillus and Lysinibacillus* genera. The most important were *B. cereus and B. licheniformis*. The most important identified strains were similar to those currently isolated in fermented traditional food suggesting at least in part food-related contaminants. The involvement of similar isolates in human infections suggests that care must be taken about their real role in sputum. The use of selective LJ with VCNT containing 10 µg of vancomycin to reduce culture contamination is a good alternative method pending the development of a more efficient method.

## Materials and Methods

### Ethics statement and biosecurity

Isolates of SFB, *M. tuberculosis* and sputum used in this study were obtained from previous study carried out in Bacteriology laboratory of Centre MURAZ research institute^12^. Briefly, these isolates were collected after culture of sputum collected at Centre regional de lutte antituberculeuse (CRLAT), without use of antiseptic mouth rinsing as described previously^12^. Sputum for VCNT evaluation were remaining samples collected from adult patients after informed consent at CRLAT during the same study^12^. This latter protocol was approved by the Institutional Ethics Committee of Centre MURAZ research institute (N° 2017-03/MS/SG/CM/CEI). All work were done under a Class 2 Biosafety Cabinets (BSC Class II) and Personal Protective Equipment (PPE) have been used as recommended by the WHO for laboratories in countries with limited resources that cultivate mycobacteria^41^.

### Subculture and morphological characterization

Fifty-tree SFB strains isolated from contaminated sputum culture on LJ media were used. Around 79% (42/53) of isolate was collected from confirmed TB cases, and 21% (11/ 53) from negative TB cases with coughing more than two weeks. These isolates, collected between February 2016 and May 2017 were stored at −20 °C in liquid media, consisting of skim milk, glycerol, glucose, and tryptone soya broth (STGG). Isolates were piqued onto 5% sheep-blood Columbia agar (Liofilchem^®^,roseta d. Abruzzi TE- Italy), and incubated for 24 hours in aerobic conditions at 37 °C. In order to obtain isolated colonies, the obtained colonies were diluted at 10^−3^ with sterile distilled water after a suspension at 0.5 McFarland, and were plated onto 5% sheep-blood Columbia agar for 24 hours in aerobic conditions at 37 °C. Isolated colonies were described, and the bacteria motility was determined after microscope observation of bacteria cells between slide and cover glass. Cells from smear of the colonies were described after Gram and Malachite green staining. Catalase test (ID color Catalase, Biomérieux SA, Lyon, Marcy-l’étoile/France) as well as the oxidase one (Oxydase test Disc, Liofilichem s.r.l Roseto D.A- Italy) were done.

### Spores forming bacteria identification

API 50 CH strip (bioMerieux, Marcy-L’Etoile, France) intended for the identification of *Bacillus* and related genera were performed according to the indications of the manufacturer. This ready-to-use medium allows the fermentation of the 49 carbohydrates and allow to identify around 29 species of *Bacillus* and related genera. A 24 hours grown bacterial colony from 5% sheep-blood Columbia agar was suspended at 2 McFarland in the CHB/E medium (bioMerieux, Marcy- l’Etoile, France) and inoculated on each strips. The carbohydrates fermentation product an acid metabolites which produce a decrease in the pH (after 24 to 48 hours of incubation at 37 °C) detected by the change in color of the indicator. The obtained biochemical profile were used for isolates identification. API test kit results were interpreted using the Analytical Profile Index (API) database of the Apiweb software (software BioMérieux, Marcy l’Etoile, France, version1.2.1). To identify an organism, the APIweb software compares the profiles obtained with those of the taxa in the database and assigns a score to each test. The strain of *Bacillus spizizenii*/ATCC/6633 was used for the internal quality control of the identification.

External quality control were done with 15 isolates identified by Matrix Assisted Laser Desorption Ionization-Time of Flight Mass Spectrometry (MALDI-TOF MS) in Marseille (France).

Grown bacterial colony from solid media was applied directly on a MALDITOF steel target plate in quadruplicate (Bruker Daltonics, Wissembourg, France) and a matrix solution was used to overlay the sample and allowed to co-crystallize. The plate was then loaded into the Microflex LT MALDI-TOF Mass Spectrometer (Bruker Daltonics) for spectra acquisition using MALDI Biotyper automation. Identification score criteria were performed as recommended by Bruker Daltonics: A score of 2.000 indicated species level identification; a score of 1.700–1.999 indicated identification to the genus level and a score of <1.700 was interpreted as misidentification.

### LJ with VCNT effectiveness on clinical sputum culture contamination

Thirty two sputum had been collected from 19 tubercular detected adult patients between December 2016 and January 2017 were used. Thirteen patients provided each 2 sputum and 6 patients provided 1 sputum. Collected sputum were stored at +4 °C−+8 °C in the refrigerator and were then transported within 72 h on ice packs to Centre MURAZ laboratory for culture processing.

After decontamination by standard methods with 4% NaOH, as previously described^41^, 100 µl of each specimen was inoculated onto 2 LJ without VCNT and 2 LJ supplemented with VCNT at10 µg of vancomycin. All inoculated media were incubated at 37 °C and exanimated at day three and seven mainly for contamination, and every week during 90 days for colonies. The criteria for contaminated culture were: (1) any change in colour or consistency of culture media, (2) development of any liquid or film on the culture media, and/or (3) presence of non-mycobacterial colonies on culture media, on the base of colony morphology and/or ZN staining.

### Mycobacteria tolerance and spores forming bacteria susceptibility to VCNT

Theses experimentations were realized between 24 November 2018 and 19 December 2018 with isolates of mycobacteria and SFB collected during a study realized between February 216 and May 2017^15^.

#### Mycobacteria tolerance to VCNT

Eight (8) isolates of mycobacteria were used including one live attenuated Bacillus Calmette Guerin strain as positive control (BCG 00002462 ref: KSV-026/17; Green Signal, India) and seven (7) clinical isolates obtained during the research activity of the mycobacteria Research Unit, Centre Muraz Bobo-Dioulasso, Burkina-Faso. In addition, sterile distilled H2O was used as negative control. The colonies gathered from LJ media, transferred to a 15-mm tube containing several glass beads (20 a 30 beads) and 3 ml of distilled water, were vortexed and a suspension calibrate to 0.5 Mac Farland using sterile H2O and a 580-nm cell densitometer (Densimat; BioMerieux, Marcy-L’Etoile, France) is realized in order to obtain a 10^6^ CFU/ml. From the 0.5 McFarland suspension, inoculum were diluted at 10^−1^ and 10^−3^ for each isolate and then, 100ul volume (containing around 10^4^ CFU for dilution at 10^−1^ and around 10^2^ CFUs for dilution at 10^−3^) were inoculated in duplicate onto LJ without or with VCNT at different concentration of vancomycin. LJ media were prepared according to manufacturer guidelines and supplemented with VCNT to make a final concentration of 10, 15 and 30 µg/ml of vancomycin (SR0091E, Oxoid Ltd, wade Road, Basingstoke, Hants,RG24 8PW, UK) before coagulating in Frio cell (Fisher Bioblok scientifique). The inoculated tubes were incubated at 37 °C and were checked visually by the naked eye every 72 h for colonies during 28 days. Ziehl-Neelsen staining of colonies was used to confirm the presence of Acid Fast Bacillus (AFB). For each isolates, colonies were counted on duplicate tubes, at deferent concentration of VCNT and the mean count were recorded.

#### Spores forming bacteria susceptibility to VCNT

Fifteen isolates including three *B. cereus*, three *B. Licheniformis*, three *Paenibacillus*, three non-reactive *Bacillus*, one *B. subtilis* one *Brevibacillus* were used. In addition, one *E. coli* ATCC25922 and sterile distilled H2O as positive and negative control respectively were used. The treatment of these isolates before inoculating onto LJ media with or without VCNT was the same as mycobacteria isolates except that beads were not used for the preparation of the bacillary suspension. The inoculated LJ with or without VCNT were checked visually by the naked eye at three, seven and fourteen days for contamination. Because of stratal contamination, CFUs counting is not possible on LJ contaminated media. Thus, we determine the contamination rate at each concentration of VCNT by reporting the ratio of SFB that have grown on LJ with VCNT on the total number of inoculated SFB at both dilution.

### Statistical analysis

Excel 2013 were used for data entry and OpenEpi 3 for data analysis. The contamination rate of clinical sputum inoculated onto LJ without or with VCNT at 10 µg of vancomycin were compared using McNemar test (2 × 2 tables) or Fischer’s exact test where appropriate. Wilson t test were used to compare mean of CFUs of LJ with VCNT at different concentration of vancomycin to control (LJ without VCNT). The proportions of SFB which have contaminated LJ with VCNT at different concentration was compared with binomial test to standard contamination rate in our laboratory (around 40%). Values of p < 0.05 were considered statistically significant.
